# Influence of multiple predators decreases body condition and fecundity of European hares

**DOI:** 10.1002/ece3.8442

**Published:** 2022-01-11

**Authors:** Martijn J. A. Weterings, Sanne Losekoot, Henry J. Kuipers, Herbert H. T. Prins, Frank van Langevelde, Sipke E. van Wieren

**Affiliations:** ^1^ Wildlife Ecology and Conservation Group Wageningen University Wageningen The Netherlands; ^2^ Wildlife Management Department of Animal Management Van Hall Larenstein University of Applied Sciences Leeuwarden The Netherlands; ^3^ School of Life Sciences Westville Campus University of KwaZulu‐Natal Durban South Africa

**Keywords:** fecundity, field metabolic rate, hunting pressure, physiology, placental scars, predator community, risk effects

## Abstract

1. We assessed the hypothesized negative correlation between the influence of multiple predators and body condition and fecundity of the European hare, from 13 areas in the Netherlands.
2. Year‐round abundance of predators was estimated by hunters. We quantified predator influence as the sum of their field metabolic rates, as this sum reflects the daily food requirements of multiple individuals. We determined the ratio between body mass and hindfoot length of hares as an index of body condition and the weight of their adrenal gland as a measure of chronic exposure to stress, and we counted the number of placental scars to estimate fecundity of hares.
3. As hypothesized, we found that the sum of field metabolic rate of predators was negatively correlated with body condition and the number of placental scars, whereas it was positively related to the weight of the adrenal glands. In contrast to the sum of the field metabolic rate, the total number of predators did not or weakly affect the investigated risk responses.
4. The sum of the field metabolic rate can be a useful proxy for the influence of multiple predators and takes into account predator abundance, type, body weight, and food requirements of multiple predators.
5. With our findings, our paper contributes to a better understanding of the risk effects of multiple predators on prey fitness. Additionally, we identify a potential contributor to the decline of European hare populations.

## Graphical abstract

Our study is one of the few field studies to correlate the potential risk imposed by multiple predators to the fecundity of a mammal prey species over multiple reproduction cycles within a year. We suggest that the sum of the field metabolic rate can be a novel index that can be easily applied to other species systems. With our findings, our paper contributes to a better understanding of the risk effects of multiple predators on prey fitness.

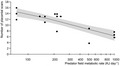

## 1 INTRODUCTION

Prey encounters with a predator can trigger anti‐predator responses (Creel, 2018) that can help prey in risky situations to escape (Sheriff et al., 2011) or hide (Weterings et al., 2016) from nearby predators. However, chronic activation of anti‐predator responses can result in increased energetic or physiological costs, which may negatively affect prey fitness (e.g., reproduction: Creel et al., 2007; Sheriff et al., 2009; survival: Griffin et al., 2011; LaManna & Martin, 2016). Chronic exposure to predation risk can also negatively affect, body condition and fecundity of prey (Hawlena & Schmitz, 2010; Zanette et al., 2014). Indeed, chronic exposure to stress is thought to directly suppress the fecundity of prey to benefit survival (or vice versa; Sinclair & Arcese, 1995). Additionally, stress effects can be passed on to the next generation through maternal effects (Boonstra et al., 1998), leading to prolonged demographic consequences.

Few prey species, however, are affected by only a single predator. For example, terrestrial ecosystems contain a high fraction of omnivores and generalist predators (Strong, 1992) that together with specialist predators can cause stress responses of prey (Frid & Dill, 2002). Additionally, human impacts, especially hunting, can elicit prey behavioral responses similar to risk associated with predators (Proffitt et al., 2009), probably with similar stress responses (Ciuti et al., 2012). Conservation of prey species can thus benefit from knowledge of multi‐predator effects (McCann, 2007). Generally, prey have 2–3 predator species preying on them (Schoener, 1989). Multi‐predator effects vary according to diet and specialization (i.e., omnivores vs. carnivores; generalists vs. specialists) and can be difficult to investigate in field situations (Schmitz, 2007). These effects critically depend on the predators' daily food requirements (Carbone & Gittleman, 2002). We use the field metabolic rate (FMR) of a potential predator species as proxy for the daily food requirements, as FMR measures an animal's total energy expenditure after all constituent costs are supported (Nagy et al., 1999). Hence, FMR could be used to represent the potential predation risk. Indeed, using FMR is proposed to be an alternative way to investigate the influence of a potential predator species on a prey species given that it may be ecologically more meaningful for potential predation risk to include predators' metabolic food requirements than predators' abundances alone (Brose et al., 2008; Nagy et al., 1999). For example, the difference between abundance and FMR may be significant when the impact of two red foxes on a prey community is compared to five least weasels. The weasels as a group have an average body weight 28 times smaller, and hence the field metabolic rate nine times smaller than that of the two foxes. Ultimately then, it is the energetic relationships between predators and prey that are important for dictating their interactions (Brose et al., 2008), and thus, the field metabolic rate of predators, as a “fundamental biological rate” (Brown et al., 2004), could link the biology of individual predators to the ecology of communities and impact predator–prey relationships (Brown et al., 2004). As such, we additionally propose the sum of the field metabolic rate (sFMR) of potential predators as a novel method to represent the potential influence of multiple predators on prey species. This implies the assumption that the contribution of each predator species can be added (i.e., substitutable) to express multiple‐predator effects on prey species (see Schmitz, 2007).

Our paper investigates the correlations between the assumed influence of multiple predators and the body condition and fecundity of a mammal prey species in a field situation, which has been done only few times. We hypothesized that higher risk from multiple predators is related to higher stress levels, and lower prey body condition and fecundity. Additionally, we investigated whether the metabolic rate of a predator would be a better predictor of risk responses compared with the absolute number of predators. We thus predicted a negative correlation between the sFMR of a multi‐predator community and body condition and fecundity of prey. Here, we study the potential effect of the predator community on a European hare (*Lepus europaeus*) population in the Netherlands. This widespread and abundant species can be found from north‐western Spain to Mongolia. While widespread, this species has experienced population declines in Europe since the 1940s (Olesen & Asferg, 2006; Smith, Jennings, & Harris, 2005). It is believed that agricultural intensification and homogenization of the European landscape (Robinson & Sutherland, 2002) has improved access of generalist predators that are thought to have negatively affected hare populations (Gorini et al., 2012; Knauer et al., 2010; Schneider, 2001).

## 2 MATERIAL AND METHODS

### 2.1 Study area

**FIGURE 1:**
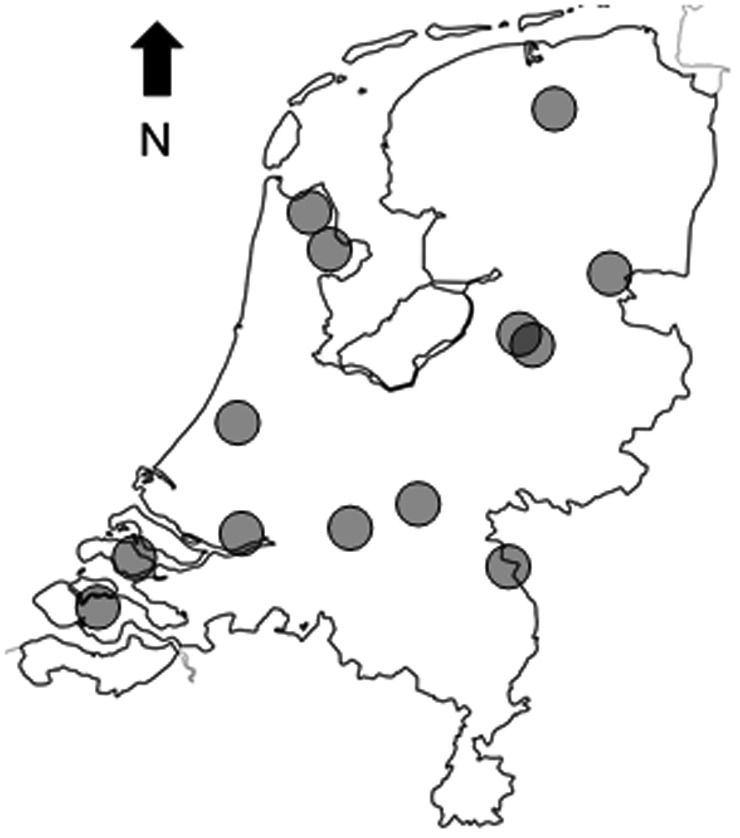
Distribution of the investigated hunting leases in the Netherlands with European hare (*Lepus europaeus*). The characteristics of hunting leases can be found in Appendix S1

We conducted the study in 13 hunting leases (mean area (SD) = 663 (551) ha; Appendix S1) distributed over the Netherlands (Figure 1). Selection of hunting leases was based on the voluntary participation of hunters in response to an invitation in the national club magazine of The Royal Dutch Hunters Association (KNJV). Hunting leases are comprised of a set of subareas on which hunting of local wildlife is managed and coordinated by a local group of hunters who together lease the local right to hunt from landowners. Subareas are homogeneous patches of vegetation types (mainly crops and pasture) or plowed areas, in human‐dominated heterogeneous landscapes.

### 2.2 Data collection

#### 2.2.1 Hare harvest and density estimation

In November and December 2013, we collected 73 hares (37 females, 35 males, 1 unknown) that were shot on 14 hunts ($\bar{X}$ ± SD = 5.6 ± 2.8 hares/hunt) within subareas in the hunting leases. Hares were hunted by hunters on foot and at fixed positions during drives. Drives consisted of a dense line (a person every 5–10 m) of hunters and beaters with or without dogs. We accompanied the hunters during the drives on clearly demarcated subareas and counted the number of hares flushed (i.e., total count) and harvested to estimate hare density and the percentage of hares shot in a hunting lease. After the hunts, we took a random subset of the total number of hares shot in a hunting lease, although in two occasions hunters removed some of the hares before we could take a sample. Hares were stored at low temperatures (<7°C) and dissected within 1–4 days ($\bar{X}$ ± SD = 1.8 ± 0.8 days) after the hunt.

#### 2.2.2 Body condition

We determined the ratio between body mass and hindfoot length of each animal (i.e., BM/HFL) as an index of body condition, because this index has been shown to be highly correlated with total bone‐marrow fat in other lagomorphs (i.e., snowshoe hares, Lepus americanus; Murray, 2002) (see Appendix S2). Additionally, we conducted a general health assessment of hares sampled before and during dissection, by assessing the presence of parasites, as well as lesions and other abnormalities that could affect body condition (Appendix S3).

#### 2.2.3 Age

We determined the weight of the eye lenses to distinguish different age classes (Peig & Green, 2010). Eye lenses were removed and stored in 10% formalin solution. After 29.6 days ± 9.1 (SD) since first storage, we air‐dried the eye lenses at 80°C for 6 days and then weighed each lens to the nearest 0.1 gram. We assigned each hare to an age class based on eye lens weight (Broekhuizen & Maaskamp, 1979) and the presence of an ulna coalescence (Stroh, 1931). Individuals with lens weight >270 mg and ulna absent were indicated as adult (>1 year), while individuals with an ulna present were indicated as subadult (≤1 year old).

#### 2.2.4 Fecundity

Female hares can have up to 5 litters each year, with a mean litter size between 2 and 3 leverets (Marboutin et al., 2003). For harvested female hares, the uteri were removed and frozen at −18°C after our dissection. We later (205.9 days ± 10.4) thawed uteri and counted the total annual number of placental scares to provide an index of the number of pregnancies as an estimator of fecundity. As uterine walls of European hare regenerate during anestrus, placental scar counts represent an index of fertilized eggs that implant during the preceding breeding season (February–August 2013). The average annual fecundity of European hares was found to be similar across regions (about 10–11 placental scars; Hackländer et al., 2011). Placental scars were counted and stained by following the protocol by Hackländer et al. (2001). The number of scars was independently assessed, discussed, and verified by Weterings and Hackländer using a 7–30× magnification zoom stereoscopic binocular.

#### 2.2.5 Weight of adrenal glands

During the lifetime of many species, the weight of the adrenal glands increases as a result of a prolonged period of exposure to stress (Harder & Kirkpatrick, 1994). We carefully removed and weighted the adrenal glands without adhering tissue as an additional estimator of stress due to chronic exposure to the potential predation risk imposed by multiple predators.

#### 2.2.6 Predator assessment

Because of the difficulty in estimating the year‐round abundance of 23 different predator species, each with their specific census methods and biases, we made use of estimates provided by hunters (see validation of hunter estimates in Appendix S5). Experienced hunters ($\bar{X}$ ± SD = 31 ± 14 years of hunting experience; Table S1) that assessed the number and type of predators in their hunting leases weekly ($\bar{X}$ ± SD = 8 ± 10 h/week; Table S1, hunter effort) were interviewed to provide estimates of the year‐round presence and abundance of 23 potential predator species of hares active on their hunting lease during the last year (Appendix S6). Potential predator species were chosen based on the literature (Tapper & Yalden, 2010) and discussions with hunters. Hares (especially when they are young) can be predated by multiple predators, such as foxes, birds of prey, and members of the mustelid family. Predation of young hares may negatively affect the condition of adult female hares via physiological pathways (Travers et al., 2010; Zanette et al., 2014).

#### 2.2.7 sFMR and hunting risk calculations

The influence of predators on prey species was expressed as the sum of the field metabolic rate (sFMR) of all potential avian and mammalian predators of hares present in a hunting lease during the year before the collection of the harvested hares. We assigned each predator to a specific predator type (i.e., all birds, Pelecaniformes, mammal omnivores, and mammal carnivores) based on Nagy et al. (1999) (Appendix S6). We then calculated the average of the lower and higher limit of the body weight for each predator species (BW_avg_; birds: Del Hoyo et al., 1992; Del Hoyo et al., 1994; Del Hoyo et al., 1996; Del Hoyo et al., 1999; Del Hoyo et al., 2009; mammals: Lange et al., 2003). The average body weight per predator species was then used in the allometric relationships of Nagy et al. (1999) to calculate field metabolic rate (FMR_BWavg_) for each predator species (per Equation 1). Finally, for birds, we calculated the proportion of the year each species was resident in the Netherlands, as many birds migrate toward southern latitudes in winter (Vogelbescherming, 2017).

Field metabolic rate (FMR) per predator species for each hunting lease (KJ day^−1^ ha^−1^) (based on Nagy et al., 1999):

(1) $$\text{FMR}=\frac{{\text{FMR}}_{{\text{BW}}_{\text{avg}}}*P}{A}$$

${\text{FMR}}_{\text{BWavg}}$ = FMR based on average body weight (KJ day^−1^), P = proportion of the year being resident (birds only), A = size of the hunting lease (ha).

#### 2.2.8 Hunting risk

We also investigated the effect of the risk of being killed by hunting on prey body condition and fecundity, to be able to assess its relative effect compared to the influence of predators, as prey responses to hunting can be stronger than responses to predators (Proffitt et al., 2009). Risk of hunting mortality was expressed as the percentage of hares shot from the total number of hares counted in a hunting lease during the hunting drives. Hunts were restricted to the period between 15 October and 31 December, with a frequency between 1 and 5 hunts per season (*n* = 8 hunting leases). We assumed that the risk of hunting mortality did not change between years, based on our communications with the local hunting groups. We thus assessed the risk of hunting mortality of the hunting period before the collection of the harvested hares.

### 2.3 Data analysis

#### 2.3.1 Model investigated

First, we investigated the correlation between the sum of the predator field metabolic rate (sFMR) and the risk of hunting mortality as predictor variables and the body condition index as response variable using a linear mixed model (LMM) in R (package lme4 version 1.1‐12; Bates et al., 2015; *n* = 66). Additionally, we investigated an alternative LMM with the total number of predators as predictor variable and the body condition index as response variable to investigate whether predator abundance better explains body condition compared to sFMR (see Appendix S4 for an overview of the global models fitted). We included the sex of the hares, their age class, and the days since the start of the data collection as fixed effects, because female hares fatten up within several weeks at the end of the year to prepare for the next breeding season (Valencak et al., 2009). Besides, body condition varies during the season (Van Vuuren & Coblentz, 1985) and scales differently between sexes (Murray, 2002). We included hunting lease as random factor, with subareas nested within hunting lease. We excluded one adult female that had a very low body weight (2416 g) compared with the rest of the adult females ($\bar{X}$ ± SD = 3642 ± 318 g).

Second, we investigated the correlations between sFMR and the risk of hunting mortality as predictor variables and the average weight of the adrenal gland as response variable using a LMM (*n* = 66). We included the age class and sex of hares as fixed effects, as adrenal glands of mammals are assumed to increase in size by chronic exposure to stress during their lifetime (Harder & Kirkpatrick, 1994). Additionally, we expected a sex‐specific stress response and perception of risk, as females have to fatten up to prepare for their first litter in winter (Valencak et al., 2009) and therefore probably respond differently to predation risk compared with males. Again, we used subareas nested within hunting lease as random factor. We excluded one adult female that had a very high average weight of the adrenal glands (0.61 g) compared with the rest of the adult females ($\bar{X}$ ± SD = 0.31 ± 0.076 g). Similarly to body condition, we also ran a model with the total number of predators as predictor variable.

Third, we investigated the correlations between the sFMR, the risk of hunting mortality, body condition, and the weight of the adrenal gland as predictor variables and the number of placental scars as response variable. Subareas nested within hunting lease were used as random factor. Correlations were investigated by fitting generalized linear mixed models in R, with a binomial error structure (*B*(*n* = 19, *p*) and logit link (*n* = 18) given that we modeled the success or failure of a fertilized egg implant in the uterus (i.e., placental scar present or absent) for each of the maximum number of possible implant locations (i.e., 19; Hackländer et al., 2001; Smith et al., 2010) in the uterus. We did not use a Poisson distribution, as this distribution did not approximate our distribution (i.e., the number of trials (*n*) multiplied by the probability of success (*p*) was much higher than 5 (NIST‐SEMATECH, 2013)). The following females were excluded from the analysis of fecundity: females with inactive uteri (i.e., uteri that were too small for reproduction after visual inspection; *n* = 13; 1 adult, 12 subadults), females with active uteri that did not reproduce (i.e., these females are possibly sterile, especially in northwest European areas, see Smith et al., 2010; *n* = 3; 1 adult, 2 subadults), and females of which the uterus contained tumors or other abnormalities (*n* = 3; 2 adults, 1 subadult). Again, we also ran a model with the total number of predators (*n* = 18) as predictor variable instead of sFMR.

We used standardized regression coefficients to assess the effect size of the predictor variables on the three response variables. Continuous predictor variables were standardized and scaled by dividing their mean by two standard deviations (Gelman, 2008). sFMR and the total number of predators were log_10_ transformed to normalize a right‐skewed distribution. Multicollinearity of continuous predictor variables was not an issue because the variance inflation factor (VIF) of all continuous predictor variables remained below 1.5 for all models. We tested the linearity between the predictors and the response variables using a generalized additive mixed model (package gamm4 version 0.2‐6). The predictors had an effective degree of freedom (edf) close to 1 and were therefore linearly related to the response variables. Model selection was performed by using the “drop1” protocol of Zuur et al. (2009) and the Akaike information criteria (AIC). The fit of the models was assessed using plots of model residuals.

## 3 RESULTS

Overall, 90.5% of the hares investigated were healthy and did not show medical abnormalities of major importance (Appendix S2).

For the metabolic rate models on body condition, the final model included sFMR, age class, and days since the start of the data collection, whereas for predator number models, the final model only included number of predators and age class (Table 1). The sum of the field metabolic rate of predators (sFMR) was negatively related to the body condition index of hares (Marginal *R* ^2^ = .61) (Table 1). Adult hares had a 21.8% higher body condition index than subadult (*p* < .001), whereas the body condition index of hares increased during the research period from autumn–winter) (*p* = .017). For the number of predators' models, while age class was correlated to body condition, the number of predators was not (Table 1). In both cases, hare sex and the percentage of hares' shot were unrelated to the body condition index.

**TABLE 1:** Final model linear mixed regression on the body condition index of European hare

No.	Final model^a^, ^b^	*n*	Variables^b^	Estimate (*β* ± ${\hat{\text{SE}}}_{\beta }$)^c^	*z*‐value	*p*‐value^d^
1	Body condition index ~ log_10_ sum field metabolic rate + AGE + DAY	66	Log_10_ sFMR	−11.4 ± 4.8	−2.4	.021*
			AGE^e^	44.7 ± 5.4	8.2	<.001***
			DAY	12.4 ± 5.0	2.5	.017*
			Intercept	205.1 ± 4.7	43.9	<.001
2	Body condition index ~ log_10_ no. of predators + AGE	66	Log_10_ tNP	−3.3 ± 5.5	−0.6	.558
			AGE^e^	46.4 ± 5.4	8.6	<.001***
			Intercept	204.7 ± 4.9	41.6	<.001

^a^ Models are based on measurements of 66 hares in 13 hunting leases collected over a period of 34 days.

^b^ Body condition index (body mass/hindfoot length; Murray, 2002), sFMR = sum of the field metabolic rate, AGE = subadult or adult, DAY = days since start of the data collection, tNP = total number of predators. The following variables were dropped out of final model 1: percentage of hares shot and sex of hares; final model 2: percentage of hares shot, sex of hares, and days since start of the data collection.

^c^ Parameters are standardized by 2 SD (Gelman, 2008).

^d^ * = *p* < .05, *** = *p* < .001.

^e^ Subadult is reference category.

For both metabolic models and predator number models on the weight of the adrenal gland, the final model included the predator index and hare sex (Table 2). The sum of the field metabolic rate of predators (sFMR) was positively related to the weight of the adrenal glands (Marginal *R* ^2^ = .14) (Table 2). The total number of predators, however, was unrelated to the weight of hare adrenal glands (Table 2). Additionally, adrenal glands of females were 0.04 ± 0.017 g ($\bar{X}$ ± SE) heavier than that of males in the model with sFMR, but not in the model with the total number of predators. In both cases, the percentage of hares shot, age class, and days since the data were collected was unrelated to the weight of the adrenal glands.

**TABLE 2:** Final model linear mixed regression on the average weight of the adrenal glands of European hare

No.	Final model^a^, ^b^	*n*	Variables^b^	Estimate (*β* ± ${\hat{\text{SE}}}_{\beta }$)^c^	*Z*‐value	*p*‐value^d^
1	Weight adrenal gland ~ log_10_ sum field metabolic Rate + SEX	66	Log_10_ sFMR	0.046 ± 0.020	2.3	.031*
			SEX^e^	0.037 ± 0.018	2.0	.046*
			Intercept	0.292 ± 0.013	22.6	<.001
2	Weight adrenal gland ~ log_10_ no. of predators + SEX	66	Log_10_ tNP	0.009 ± 0.024	0.4	.771
			SEX^e^	0.033 ± 0.018	1.8	.072^#^
			Intercept	0.293 ± 0.014	21.2	<.001

^a^ Models are based on measurements of hares in 13 hunting leases collected over a period of 34 days.

^b^ sFMR = sum of field metabolic rate, SEX = male or female, tNP = total number of predators. The following variables were dropped out of final Models 1 and 2: percentage of hares' shot, age class, and days since start of the data collection.

^c^ Parameters are standardized by 2 SD (Gelman, 2008).

^d^ # = *p* < .1, * = *p* < .05, *** = *p* < .001.

^e^ Male is reference category.

**FIGURE 2:**
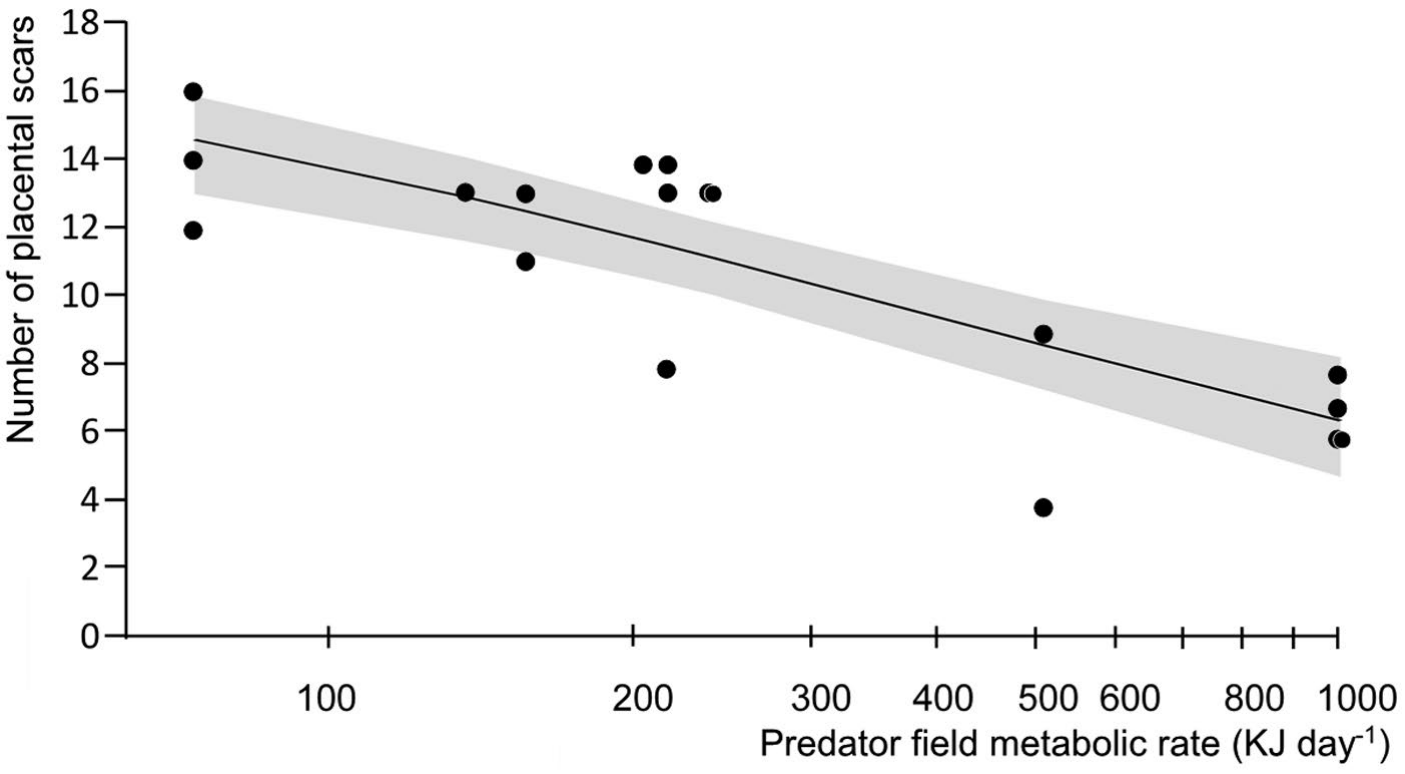
The relationship between the sum of the field metabolic rate of predators and number of placental scars of European hare (*Lepus europaeus*). The sum of the field metabolic rate of predators is a proxy of the influence of multiple predators on prey species. Dots are the raw data points, *n* = 18; line = marginal effects of predicted probabilities of binomial model (±95% CI, *Z* = −5.3, df = 17, *r* ^2^ = .65). Note the logarithmic scale of the *x*‐axis

For both metabolic models and the predator models on the number of placental scars, the final model only included the predator index (Table 3). The number of placental scars of hares was strongly negatively correlated with the sFMR of predators (Nagelkerke pseudo‐*R* ^2^ = .82; Table 3; Figure 2). The total number of predators was weakly negatively correlated to the number of placental scars (Nagelkerke pseudo‐*R* ^2^ = .23; Table 3). In both cases, the percentage of hares' shot, the body condition index, and the weight of the adrenal glands had no correlation with the number of placental scars.

**TABLE 3:** Results of generalized linear mixed models on the number of placental scars of European hare

No.	Final model^a^, ^b^	*n*	Variables^b^	Estimate (*β* ± ${\hat{\text{SE}}}_{\beta }$)^c^	*Z*‐value	*p*‐value^d^
1	No. of placental scars ~ log_10_ sum field metabolic rate	18	Log_10_ sFMR	−1.3 ± 0.2	−5.3	<.001***
			Intercept	0.3 ± 0.1	2.5	.011
2	No. of placental scars ~ log_10_ no. of predators	18	Log_10_ tNP	−0.5 ± 0.2	−2.1	.033*
			Intercept	0.3 ± 0.1	2.9	.003

^a^ Models are based on measurements of hares in 7 hunting leases collected over a period of 34 days.

^b^ sFMR = sum of field metabolic rate, tNP = total number of predators. The following variables were dropped out of final Model 1: percentage of hares shot, body condition index of hares, the weight of the adrenal gland; final Model 2: body condition index of hares, the weight of the adrenal gland.

^c^ Parameters are standardized by 2 SD (Gelman, 2008).

^d^ * = *p* < .05, *** = *p* < .001.

**FIGURE 3:**
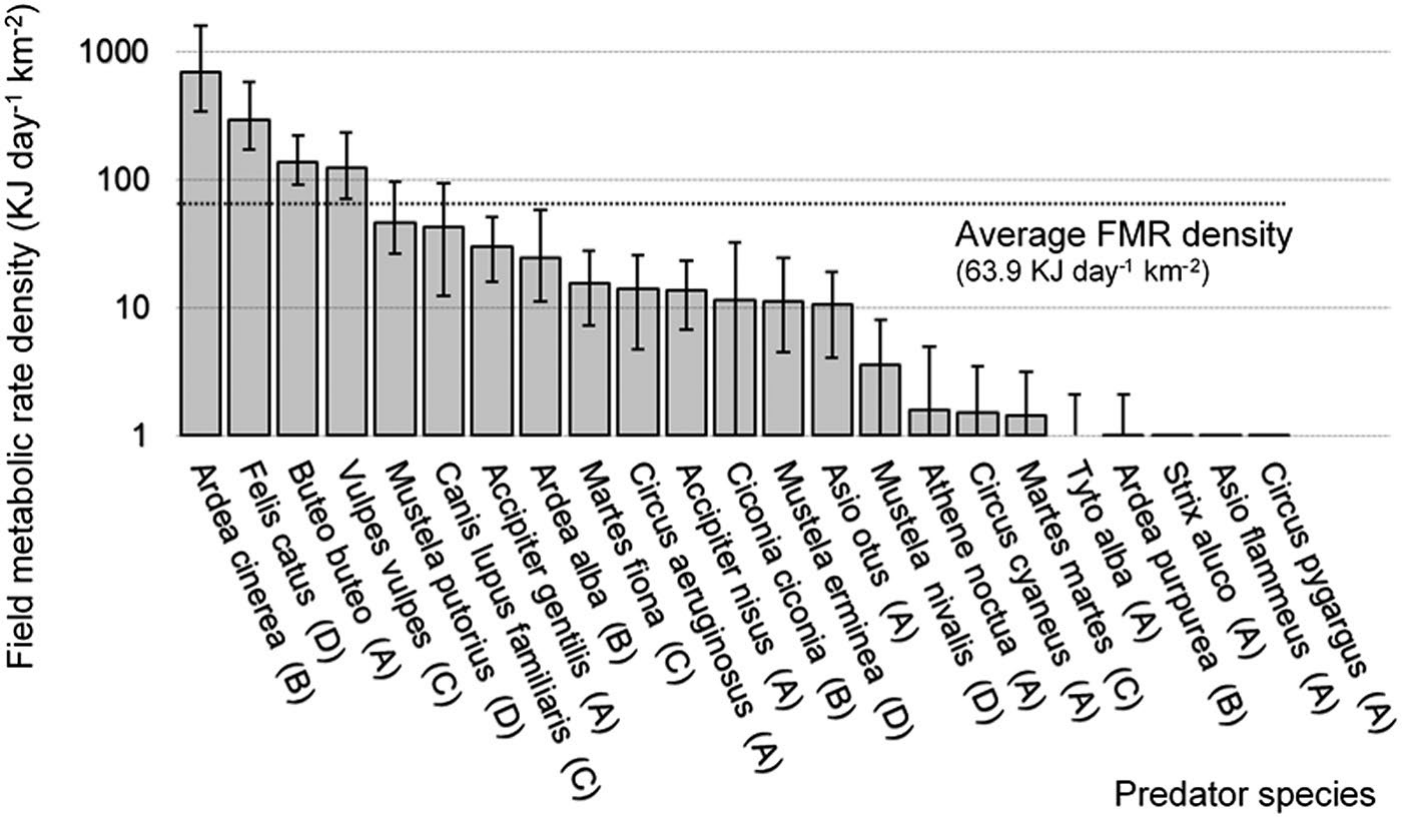
Field metabolic rate density ($\bar{X}\pm \text{SE}$) for predators (*n* = 23) of European hare (*Lepus europaeus*) in Dutch hunting leases (*n* = 13). Predator types based on Nagy et al. (1999): A = all birds, B = Pelecaniformes, C = mammal omnivores, D = mammal carnivores. Field metabolic rate density = weighted species density * average species field metabolic rate (i.e., a measure of predator influence on prey species). The weighted species density = estimates of species abundance provided by hunters weighted by the size of the hunting lease and multiplied by the proportion of the year that the species is present (see text for further explanation). Note the logarithmic scale of the *y*‐axis

Four predator species had an above average FMR density (>63.9 KJ day^−1^ ha^−1^) in the hunting leases investigated, namely gray heron (*Ardea cinerea*), domestic or feral cat (*Felis catus*), Eurasian buzzard (*Buteo buteo*), and red fox (*Vulpes vulpes*) (Figure 3, Appendix S6).

## 4 DISCUSSION

Our study is one of the few field studies to correlate the potential risk imposed by multiple predators to the fecundity of a mammal prey species over multiple reproduction cycles within a year. Additionally, we show that irrespective of the predator index used, there was a negative correlation between fecundity and predation risk. Our findings are in line with other studies that have demonstrated relationships between predation risk and fecundity of animals (birds: Zanette et al., 2011; mammals: Monclús et al., 2011; Sheriff et al., 2009, 2010, 2011), a relationship which is difficult to measure in the field. Indeed, Hawlena and Schmitz (2010) and Zanette et al. (2014) reviewed 81 studies that investigated effects of predation risk on species behavior, physiology, or reproduction. The majority of these studies (>86%) focused on a single reproduction cycle and involved the manipulation of (field) conditions or the capture of individuals (but see Monclús et al., 2011). Manipulations are often necessary given that monitoring the fecundity of crepuscular mammalian species, especially non‐central place foragers, is nearly impossible (Sheriff et al., 2009). However, given that results may be an artifact of laboratory conditions (e.g., Mappes et al., 1998), it is always good to validate that these relationships do occur in the wild.

Changes in physiology, especially due to glucocorticoids (Sheriff et al., 2009), can explain the influence of predators on fecundity (Hawlena & Schmitz, 2010) when investigated at the appropriate timescale (Corlatti et al., 2014). It is thought that animals with poor body condition may reflect selection for low‐risk environments with little nutritional value (Heithaus et al., 2007) and that body condition should ultimately affect survival or reproduction (Sinclair & Arcese, 1995). However, we show that the body condition index was not related to the number of placental scars. It is possible this is because body condition was measured on (and varies over) a short timescale, while any effect on fecundity should be relevant over a longer timescale (Corlatti et al., 2014). For example, body condition can be related to periods of adverse weather (van Wieren et al., 2006) or temporal variation in body weight (Van Vuuren & Coblentz, 1985). In accordance to this, our analysis showed that the body condition was affected by seasonal effects. In contrast, our number of placental scars represents an index of the total number of fertilized eggs that implant during a much longer period, that is, the breeding season between February and August. Similar to the body condition, the adrenal glands can show seasonal trends in body mass (McCreedy & Weeks, 1992). Besides, variation in the concentration of sodium (Na) between sandy and clayey coastal soils in the Netherlands could affect the size of the adrenal glands (McCreedy & Weeks, 1992) making it less suitable as a proxy for predation risk. Food availability has also been found to affect fecundity (Zanette et al., 2014), whereas disease and parasites have been investigated earlier but were not found to affect fertility (Krebs et al., 2001; Murray et al., 1998). Similarly, in our study, we did not find any indication that diseases and parasites were of such an importance that they could explain a reduction in fecundity.

In accordance with Harder and Kirkpatrick (1994), we found that the weight of the adrenal gland as a measure of chronic exposure to stress was correlated with the sFMR of predators, however weakly. Our results showed that females had larger adrenal glands compared with males. Because female hares are capital breeders that build up fat reserves during the winter period (Valencak et al., 2009), therefore, they respond maximally to predation risk (Luttbeg et al., 2003) and could perceive higher levels of stress that result in larger adrenal glands.

The use of sFMR, on the relevant temporal scale, could be a promising novel method to investigate multi‐predator effects on the body condition and fecundity of prey. We estimated predation risk by the sFMR of predators reported by experienced hunters from hunting leases. As shown by our results, sFMR as an index of predator influence can be a useful index over predator abundance (e.g., see Monclús et al., 2009; Monclús et al., 2011; Sheriff et al., 2009), given that it was related to each of the metrics we examined. This is likely, because sFMR integrates the variation in predator abundance, type, and body weight to reflect the daily food requirements of all predators (Carbone & Gittleman, 2002). Hunter estimates seemed to be a valid metric as they were strongly positively correlated with independent data on predator species distribution (Gaston & Blackburn, 2000; see Appendix S5). Nevertheless, it is difficult to find support for our initial assumption that the contribution of each predator species is substitutable. The sFMR was strongly correlated to the body condition and the fecundity of prey, even though the predator community composition was different in each hunting lease. Multiple‐predator effects on prey species are thought to be substitutable if the potential predators in general segregate their habitat, while the prey species would make use of a wide range of different habitats (Schmitz, 2007). Further research should explore the relevance for sFMR as proxy for the potential predation risk of multiple predators.

There are several factors which may affect the relationships we documented here. First, the presence of other prey species will affect the diversity and abundance of predators (Carbone & Gittleman, 2002). Changes in prey and predator community composition will alter various risk‐associated relationships (Duffy et al., 2007). Especially, the presence of predators with a large average body weight and a high abundance may result in a high year‐round predation risk for the prey. Second, effects of predators on prey species (i.e., risk perception) depend on hunting mode, habitat use (Schmitz, 2007), interactions with other predators (Vance‐Chalcraft & Soluk, 2005), resource specificity (Duffy et al., 2007), and prey risk detection (Monclús et al., 2009) that can vary during the course of the season. Finally, predation risk of prey depends on their life stage. For example, young hares are affected by a wider variety of predators, with different risk responses, than adult hares and these effects may translate to changes in fecundity later in life. Besides, predation of young may affect the condition of adult females in species with multiple breeding attempts (Travers et al., 2010; Zanette et al., 2014). Nevertheless, even species that disturb hares can trigger anti‐predator behavior (Frid & Dill, 2002). Finally, our study assessed non‐randomly selected hunting leases, involved few samples of uteri, and only spanned a single hunting season. It is possible that these relationships change as a function of where animals are in their geographic range or that these relationships are an artifact of small sample sizes over limited time frames, all of which could be investigated in future research.

Our paper reports negative correlations between the assumed influence of multiple predators and the body condition, the weight of the adrenal gland and the fecundity of a mammal prey species in the wild. We suggest that the sum of the field metabolic rate, which takes into account predator abundance, type, body weight, and food requirements of multiple predators, can be a useful novel index that can be easily applied to other systems. With our findings, our paper contributes to a better understanding of the influence of multiple predators on prey species fitness to benefit conservation. Intensification of agriculture and homogenization of the landscape (i.e., a loss of habitat diversity, structure, and quality) strongly affected hare populations in north‐western Europe (Smith, Jennings, & Harris, 2005). However, predator numbers in this region have increased in the last decades (e.g., birds of prey: Parlevliet, 2003; red fox: Tapper, 1992; Knauer et al., 2010), while predators have also expanded their distribution (e.g., birds of prey: Boele et al., 2008; Hustings & Vergeer, 2002; red fox: Davidson et al., 2012). This study showed that a twofold increase in predator field metabolic rate could reduce the fertility of hares by about 16%. It thus supports the idea that the predator community negatively affects the population dynamics of European hare (see Smith, Jennings, & Harris, 2005), which may also explain their decline during the last decades (Knauer et al., 2010).

## CONFLICT OF INTEREST

None.

## AUTHOR CONTRIBUTIONS

**Martijn J. A. Weterings:** Conceptualization (lead); Data curation (lead); Formal analysis (lead); Funding acquisition (lead); Investigation (lead); Methodology (lead); Project administration (lead); Supervision (lead); Validation (lead); Writing – original draft (lead); Writing – review & editing (equal). **Sanne Losekoot:** Conceptualization (supporting); Data curation (supporting); Investigation (equal); Methodology (supporting); Project administration (supporting); Writing – original draft (supporting); Writing – review & editing (equal). **Henry J. Kuipers:** Conceptualization (supporting); Formal analysis (equal); Methodology (supporting); Supervision (supporting); Validation (supporting); Writing – original draft (supporting); Writing – review & editing (equal). **Herbert H. T. Prins:** Funding acquisition (supporting); Resources (supporting); Supervision (supporting); Writing – review & editing (equal). **Frank van Langevelde:** Conceptualization (supporting); Methodology (supporting); Supervision (supporting); Writing – original draft (supporting); Writing – review & editing (equal). **Sipke E. van Wieren:** Conceptualization (supporting); Funding acquisition (supporting); Methodology (supporting); Supervision (supporting); Writing – original draft (supporting); Writing – review & editing (equal).

## Supporting information

Appendix S1‐S6

## Data Availability

The data are deposited in the Dryad Digital Repository (https://doi.org/10.5061/dryad.2jm63xsqp). The use of dead hares, which were not killed for this study but made available by hunters from their bag, was not part of an animal experiment as referred to the Dutch Act on Animal Experiments. An ethical assessment was therefore not needed. This was confirmed by the local animal welfare officer of the Wageningen University Animal Experiment Committee.
