# Supplementary material for: Influence of multiple predators decreases body condition and fecundity of European hares
Source: Ecol Evol. 2022 Jan 11;12(1):e8442. doi: 10.1002/ece3.8442 (PMC8809432; doi:10.1002/ece3.8442)
Supplement: Supplementary file 1 — Appendix S1‐S6 [file ECE3-12-e8442-s001.docx]

**Appendices**

Appendix S1

Table S1: Characteristics of Dutch hunting leases (n = 13)

| Hunting lease number | Number of subareas | Number of hunts | Area size  (ha) | Hunter ^1^  experience  (year) | Hunter effort ^2^  (h week^-1^) | European hare  density  (ha ^-1^) | Predator community  species composition ^3^ | Total number of predators estimated by hunters | Sum of Field Metabolic Rate  (KJ day^-1^ ha^-1^) |
| --- | --- | --- | --- | --- | --- | --- | --- | --- | --- |
| 1 | 2 | 1 | 1375 | 25 | 10 | 0.22 | 1, 2, 4, 6, 7, 12, 13, 18, 19, 20, 21, 22, 23 | 121 | 145.1 |
| 2 | 2 | 1 | 2000 | 57 | 40 | - | 1, 2, 4, 5, 6, 7, 8, 11, 12, 13, 14, 15, 17, 19, 20, 21, 22, 23 | 229 | 126.8 |
| 3 | 1 | 1 | 100 | 60 | 8 | 0.57 | 1, 2, 6, 7, 8, 12, 13, 15, 19, 20, 21, 22, 23 | 67 | 1076.0 |
| 4 | 1 | 1 | 600 | 26 | 4 | 0.72 | 1, 2, 6, 12, 13, 17, 19, 20 | 23 | 66.8 |
| 5 | 1 | 1 | 1000 | 26 | 8 | 0.05 | 1, 2, 6, 7, 11, 13, 16, 20, 21, 22, 23 | 82 | 125.9 |
| 6 | 3 | 1 | 260 | 24 | 2 | 0.31 | 2, 6, 7, 8, 11, 12, 13, 20, 21, 22, 23 | 67 | 226.3 |
| 7 | 3 | 1 | 750 | 25 | 10 | - | 2, 6, 7, 8, 11, 13, 19, 20, 21, 22, 23 | 36 | 49.6 |
| 8 | 3 | 1 | 850 | 30 | 5 | 0.46 | 2, 3, 4, 5, 6, 7, 11, 12, 13, 14, 16, 19, 20, 21, 22, 23 | 158 | 397.0 |
| 9 | 6 | 2 | 150 | 9 | 5 | 0.34 | 1, 2, 5, 6, 10, 11, 12, 13, 15, 16, 18, 19, 20, 22, 23 | 77 | 998.6 |
| 10 | 2 | 1 | 650 | 25 | 2.5 | 0.08 | 1, 4, 6, 7, 10, 12, 13, 15, 19, 20 | 58 | 193.7 |
| 11 | 2 | 1 | 450 | 32 | 2 | 0.17 | 1, 6, 9, 11, 12, 13, 17, 19, 20, 21, 23 | 60 | 238.5 |
| 12 | 3 | 1 | 180 | 35 | 5 | 0.46 | 1, 2, 5, 6, 7, 8, 11, 12, 13, 17, 19, 20, 22, 23 | 34 | 204.7 |
| 13 | 3 | 1 | 250 | 30 | - | 0.30 | 1, 2, 4, 5, 6, 11, 12, 13, 15, 19, 20, 21, 22, 23 | 80 | 497.3 |

^1^ Hunter experience = the number of years a hunter has been hunting on the hunting lease;  ^2^ Hunter effort = the number of hours per week a hunter spends in the hunting lease;  ^3^ Predator community species composition: 1 *Accipiter gentilis,* 2 *Accipiter nisus*, 3 *Asio flammeus,* 4 *Asio otus*, 5 *Athene noctua*, 6 *Buteo buteo,* 7 *Circus aeruginosus*, 8 *Circus cyaneus*, 9 *Circus pygargus,* 10 *Strix aluco,* 11 *Tyto alba*, 12 *Ardea alba,* 13 *Ardea cinerea*, 14 *Ardea purpurea*, 15 *Ciconia ciconia*, 16 *Canis lupus familiaris*, 17 *Martes fiona,* 18 *Martes martes,* 19 *Vulpes vulpes,* 20 *Felis catus,* 21 *Mustela ermine,* 22 *Mustela nivalis*, 23 *Mustela putorius*

Appendix S2

Table S2: An overview of European hare morphometric measurements $(\bar{X}\pm SD)$ per sex and age class, shot by hunters in the Netherlands.

| Sex | Male | | | | Female | | | |
| --- | --- | --- | --- | --- | --- | --- | --- | --- |
| Age class ^1^ | Sub adult | n | Adult | n | Sub adult | n | Adult | n |
| Body weight (g)  Hind foot length (avg.)(cm)  Adrenal weight (avg.)(mg)  Eye-lens weight (avg.)(mg) ^2^  Placental scars  n = 0  abnormalities  n > 0 & no abnormalities | 3250 ± 502  14.4 ± 0.43  280 ± 52.8  197 ± 43.8 | 13  13  13  13 | 3663 ± 276  14.6 ± 0.71  301 ± 77.0  327 ± 30.2 | 22  22  22  22 | 3175 ± 559  14.3 ± 0.58  291 ± 67.2  201 ± 34.4  0 ± 0  0 ± 0  10.0 ± 5.20 | 17  17  17  18  2  1  3 | 3593 ± 420  14.6 ± 0.48  364 ± 102  325 ± 31.0  0 ± 0  0.5 ± 0.71  10.9 ± 3.31 | 19  19  18  19  1  2  15 |

^1^ Age class is based on eye lens weight (Broekhuizen and Maaskamp 1979) and the presence of an ulna coalescence (Stroh 1931). Individuals with a lens weight > 270 mg and ulna absent were indicated as adult (> 1 year), while individuals with an ulna present were indicated as sub adult (≤ 1 year old).

^2^ Protocol by Broekhuizen and Maaskamp (1979), eye-lens weight air dried at 80°C for 6 days

Appendix S3

*Hare health assessment*

Tissue samples of liver, lung, spleen, brain, heart and kidneys were examined by the Dutch Wildlife Health Centre (DWHC) for general pathology. Our general health assessment indicated that in 7.5% of the carcasses investigated no abnormalities were found. 83% of the hares had medical abnormalities of minor importance, of which nephritis in kidneys (n = 17), inflammatory infiltrates (n = 15), hepatitis (n = 8) and pneumonia (n = 7) were most often found. We found that 9.5% of the hares had medical abnormalities greater than minor importance, such as liver flukes (n = 3), pseudotuberculosis (n = 2) and encephalitis (n = 2). Only two ecto-parasites (i.e., ticks only) were encountered on two different individuals.

Appendix S4

Table S4: An overview of the global models fitted.

| Model no. | Type ^1^ | n | Model ^2^ | Random factor |
| --- | --- | --- | --- | --- |
| 1 | LMM | 66 | 1. Body condition Index ~ Log_10_ sFMR + PS + SEX + AGE + DAY 2. Body condition Index ~ Log_10_ tNP + PS + SEX + AGE + DAY | lease/ sublease  (nested) |
| 1 | LMM | 66 | 1. Weight adrenal gland ~ Log_10_ sFMR + PS + SEX + AGE 2. Weight adrenal gland ~ Log_10_ tNP + PS + SEX + AGE | lease/ sublease  (nested) |
| 3 | GzLMM | 18 | 1. Number of placental scars ~ Log_10_ sFMR + PS + BCI + WAG 2. Number of placental scars ~ Log_10_ tNP + PS + BCI + WAG | lease/ sublease  (nested) |
| 4 | GzLMM | 320 | 1. Number of predators estimated by hunters ~ SD + P | Area & species |

^1^ LMM = Linear mixed model, GzLMM = Generalized linear mixed model; ^2^ sFMR = sum of field metabolic rate of predators, tNP = total number of predators, PS = percentage of hares shot, SEX = male or female, AGE = sub adult or adult, DAYS = days since start of the research, SD = species distribution, i.e., fraction of grid cells occupied by a species, P = proportion of the year a species is resident.

Appendix S5

*Validation of hunter estimates*

We assessed the validity of estimates of predator abundance provided by the hunters, using independently collected data from Dutch mammal (NDFF 2015) and bird species distribution maps (SOVON 2017). As species abundance is positively correlated to species distribution in space (Gaston & Blackburn 2000), we counted for each predator species the fraction of 5x5 km^2^ grid cells of the national Dutch species monitoring grid that were occupied in a 15x15 km^2^ grid that encompassed and surrounded the hunting leases.

We correlated the estimates of predator abundance provided by hunters with predator species distribution (i.e., the faction of grid cells occupied by a predator species on and around the hunting lease) and the proportion of the year predators are resident. We used standardized regression coefficients to assess the effect of the predictor variables on the response variable. Continuous predictor variables were standardized and scaled by dividing their mean by two standard deviations (Gelman 2008). Multicollinearity of continuous predictor variables was not an issue, because the Variance Inflation Factor (VIF) of all continuous predictor variables remained below 1.5. We fitted Generalised linear mixed models with a Poisson distribution and log-link function in R (package lme4 version 1.1-12), with predator species and hunting lease as a random factor. Model selection was performed by using the ‘drop1’ protocol of Zuur et al. (2009) and the Akaike Information Criteria (AIC). The fit of the models was assessed visually using plots of model residuals.

*Results*

Estimates of predator abundance provided by hunters were positively correlated with predator species distribution (Table A5). Estimates of predator abundance provided by hunters were not correlated with proportion of the year a predator was present.

Table S5: Results of the Generalized linear mixed models on the total number of predators of European hare estimated by hunters.

| No. | Final model ^1,2^ | n | Variables | Estimate (β$\pm{\hat{\mathrm{SE}}}_{\beta}$) ^3^ | Z value | P value ^4^ |
| --- | --- | --- | --- | --- | --- | --- |
| 1 | Total number of predators ~ Species distribution + proportion of year predator was resident | 320 | Species distribution | 1.3 $\pm$ 0.1 | 9.8 | < 0.001 *** |
|  |  |  | Proportion of year predator was resident | 1.0 $\pm$ 0.6 | 1.8 | 0.08 # |
|  |  |  | Intercept | 0.01 $\pm$ 0.3 | 0.03 | 0.98 |

^1^ Models are based on 10-13 estimates of each of the 25 predators in 13 hunting leases, random factor: area type & predator species; ^2^ Species distribution was quantified as the faction of grid cells occupied by a predator species on and around the hunting lease; ^3^ parameters are standardized by 2 SD (Gelman, 2008); ^4^ # = p < 0.1, *** = p < 0.001.

Appendix S6

Table S6: Characteristics of predator species of European hare in Dutch study sites (n = 23)

| Nr. | Species | Common name | Weighted density ^1^  (100 km^-2^)  $\bar{X}$ (min. – max.) | Predator type ^2^ | Average body weight ^3^  (g) | Field metabolic rate ^2^  (KJ day^-1^)  $\bar{X}$ (95% CI) | Field metabolic rate density ^4^  (KJ day^-1^ km^-2^)  $\bar{X}$ (95% CI) |
| --- | --- | --- | --- | --- | --- | --- | --- |
| 1 | *Accipiter gentilis* | Northern goshawk | 2.6 (0 – 13.9) | A | 1013 | 1170 (545 – 2512) | 30 (2 –72) |
| 2 | *Accipiter nisus* | Eurasian sparrow hawk | 3.2 (0 – 18.4) | A | 226 | 421 (197 – 900) | 14 (0 – 33) |
| 3 | *Asio flammeus* | Short-eared owl | < 0.1 (0 – 1.5) | A | 350 | 567 (265 – 1214) | 0.2 (0 – 1.4) |
| 4 | *Asio otus* | Long-eared owl | 2.0 (0 – 13.9) | A | 328 | 542 (253 – 1160) | 10.6 (0 – 27) |
| 5 | *Athene noctua* | Little owl | 0.4 (0 – 11.6) | A | 184 | 366 (171 – 783) | 1.6 (0 – 8.2) |
| 6 | *Buteo buteo* | Eurasian buzzard | 12.2 (2.3 – 26.7) | A | 945 | 1115 (519 – 2394) | 136 (47 – 301) |
| 7 | *Circus aeruginosus* | Western marsh harrier | 1.7 (0 – 12.2) | A | 603 | 821 (383 – 1760) | 14 (0 – 37) |
| 8 | *Circus cyaneus* | Hen harrier | 0.2 (0 – 2.8) | A | 440 | 663 (309 – 1419) | 1.5 (0 – 5.3) |
| 9 | *Circus pygargus* | Montagu’s harrier | < 0.1 (0 – 0.3) | A | 336 | 552 (258 – 1181) | 0.02 (0 – 0.3) |
| 10 | *Strix aluco* | Tawny owl | < 0.1 (0 – 0.9) | A | 497 | 720 (336 – 1542) | 0.3 (0 – 1.3) |
| 11 | *Tyto alba* | Barn owl | 0.2 (0 – 1.7) | A | 321 | 535 (250 – 1144) | 1.0 (0 – 3.1) |
| 12 | *Ardea alba* | Great egret | 1.5 (0 – 8.0) | B | 1100 | 1675 (478 – 5863) | 25 (0 – 90) |
| 13 | *Ardea cinerea* | Grey heron | 24.4 (1.2 – 116.1) | B | 2037 | 2817 (787 – 10082) | 687 (10 – 2517) |
| 14 | *Ardea purpurea* | Purple heron | < 0.1 (0 – 1.2) | B | 935 | 1460 (411 – 5192) | 0.6 (0 – 3.6) |
| 15 | *Ciconia ciconia* | White stork | 0.3 (0 – 2.3) | B | 3350 | 4287 (1077 – 17069) | 11 (0 – 52) |
| 16 | *Canis lupus familiaris* | Domestic dog | 0.6 (0 – 4.6) | C | 31500 | 6764 (2222 – 20584) | 42 (0 – 143) |
| 17 | *Martes Fiona* | Beech marten | 1.9 (0 – 9.3) | C | 1400 | 819 (352 – 1904) | 15 (0 – 40) |
| 18 | *Martes martes* | European pine marten | 0.2 (0 – 2.3) | C | 1375 | 809 (349 – 1879) | 1.4 (0 – 4.9) |
| 19 | *Vulpes vulpes* | Red fox | 5.2 (0 – 18.6) | C | 6750 | 2380 (906 – 6255) | 123 (20 – 336) |
| 20 | *Felis catus* | Domestic or feral cat | 10.8 (2.3 – 29.0) | D | 4250 | 2707 (924 – 7927) | 292 (57 – 872) |
| 21 | *Mustela ermine* | Stoat | 4.0 (0 – 26.7) | D | 293 | 278 (88 – 878) | 11 (0 – 38) |
| 22 | *Mustela nivalis* | Least weasel | 3.4 (0 – 15.1) | D | 95 | 107 (32 – 357) | 3.6 (0.2 – 12) |
| 23 | *Mustela putorius* | European polecat | 5.6 (0 – 20.9) | D | 1050 | 825 (274 – 2483) | 46 (7.2 – 143) |

^1^ Predator abundances estimated by hunters, weighted by the size of the hunting lease and multiplied by the proportion of the year that the species is present; ^2^ Based on Nagy et al. (1999): A = all birds, B = Pelecaniformes, C = mammal omnivores, D = mammal carnivores; ^3^ Average of lower and higher limit of body weight, birds: Del Hoyo et al. (1992; 1994; 1996; 1999; 2009); mammals: Lange et al. (2003); ^4^ Field metabolic rate density = weighted species density * average species field metabolic rate (n = 13)
